# Correlates of patient satisfaction with physician visit: Differences between elderly and non-elderly survey respondents

**DOI:** 10.1186/1477-7525-5-62

**Published:** 2007-11-24

**Authors:** Meg C Kong, Fabian T Camacho, Steven R Feldman, Roger T Anderson, Rajesh Balkrishnan

**Affiliations:** 1 Department of Pharmacy Practice and Administration, Ohio State University College of Pharmacy, Columbus, Ohio, USA; 2 Division of Public Health Sciences, Wake Forest University School of Medicine, Winston Salem, North Carolina, USA

## Abstract

**Background:**

Few studies document differences in patient satisfaction with physicians in the elderly (≥ 65 years) and compare it to non-elderly (<65 years) patients.

**Methods:**

A cross-sectional survey study on a convenience sample of 20,901 patients rated their recent visit to a physician through a web-based survey. Survey included validated questions based on aspects of physician care practice such as "friendliness", wait times and time spent with doctor. These scales were then used to measure patient satisfaction with physician. Statistical analysis involved pair-matching of non-elderly patients with elderly patients (both cohorts, n = 507 each) using propensity scores.

**Results:**

Even though elderly and non-elderly patients had similar waiting times, elderly patients gave higher physician satisfaction scores than non-elderly patients (all p < 0.05). When predictors of physician satisfaction ratings were examined, shorter waiting time was more significantly associated with better treatment satisfaction in non-elderly patients (partial rho = -0.25 in the non-elderly compared to partial rho = -0.11 in elderly, p < 0.05). Increased time spent with the physician was more significantly correlated with higher physician satisfaction ratings in the non-elderly patients (partial rho = 0.38 in the non-elderly compared to partial rho = 0.18, p < 0.001).

**Conclusion:**

Increased patient satisfaction ratings of the non-elderly were associated more strongly with shorter waiting times than in the elderly. However overall, elderly patients reported similar waiting times and better physician satisfaction scores. Similarly, higher physician satisfaction in non-elderly patients were more strongly associated with increased time spent with physician than in the elderly patients.

## Background

The modern era has seen a population-wide rise in the average life expectancy. It follows then that the population of people who live to the age of 65 and older is also rising [1], for example, the so called 'baby boomers'. Problems and trends particularly associated with this age group are thus ones of growing importance, and deserve special attention and consideration. Notably, elderly patients have disproportionately high need for and usage of health care. For example, it has been found that most Medicare recipients suffer more than one chronic disease, and seek active physician care for treatment of their medical conditions [2]. So, when the increase in the elderly population is considered together with their higher utilization of health care it becomes clear that examining quality of health care provided to older adults is a relevant and important topic.

Our primary interest in this study was the perceived satisfaction of patients visiting their physicians, and in particular the differences between those aged 65 years and older and the rest of the population. It has been shown in the literature that patient satisfaction ratings can be a key indicator of quality of care [3]. Patient satisfaction itself has been shown to be influenced by a number of variables, such as waiting time, time spent with physician, convenience of office, and attitude and demeanor of physician, among many others [3-7]. Survey data may be used to discern the degree to which such variables correlate with patient satisfaction in particular populations, and thus what areas may best improve quality of care. However, there is scant literature examining the specific differences in the correlates of patient satisfaction with a physician visit between elderly and non-elderly patients. Through analysis of satisfaction rating data, this study aims to be a preliminary examination of the differences on the correlates of patient satisfaction between elderly and non-elderly patients.

## Methods

The data for this study was collected from responses to an on-line survey (conducted through DrScore.com) which was both national and cross-sectional. In order to produce data for patient advocacy research and patient satisfaction report cards for physicians, the survey gathered anonymous patient ratings of physicians in the United States (U.S.).

Survey questions were directed at the most recent outpatient visit, and allowed patients to find their doctor on a list of U.S. physicians. Participation was advertised in three ways: a public radio show (The Peoples Pharmacy), by patient advocacy groups, and through on-line search engines. The patients who participated were asked by the survey to both rate their physician on several dimensions of health care experience and provide specific comments as to what they found exceptional or in need of improvement. Responses to the questions were on a scale from 0 ("not at all satisfied") to 10 ("extremely satisfied"). Overall satisfaction scores were assessed in this study using provider ratings ("Physician Care", 9 items) on the thoroughness of care, physician communication and follow-up, listening, demeanor, discussion of test results, answering questions, treatment success, and including the patient in the decision processes. The summed scores were scaled to the range of 0 to 100 by taking each item mean and multiplying it by 100, representing complete satisfaction on all characteristics measured.

In order to measure patient waiting time, the patient was asked to recall the amount of time he or she had waited before being seen by their physician at a scheduled appointment, and then choose the appropriate category. The response categories were: 1–5 minutes waiting time, 6–15 minutes, 16–30 minutes, 31–60 minutes, and over 1 hour. Shorter time intervals were chosen for the first 30 minutes because pilot data showed that roughly 70% of responding patients waited less than 15 minutes. Perceived time spent with their physician was similarly measured by patient recall, with the following response categories: less than 5 minutes, 6–10 minutes, and greater than 10 minutes. The measures of reliability (.95) and validity (.99) of the survey questions used have been established and described elsewhere [8,9].

No personal identifying information was collected (such as name or address) and expedited Institutional Review Board approval was obtained to analyze the non-identifying data from Wake Forest University School of Medicine.

### Data Analysis

In the original convenience sample of 20,902, only 523 respondents were aged 65 years and older. The elderly group (n = 523) to the non-elderly group (n = 20379) approximate ratio of 1:39 allowed us to calculate propensity scores to control for demographic variables. This technique helps control for unobserved selection bias in responses and increase precision [10]. Patients who were younger than 65 years (n = 507) were pair-matched with those who were 65 years and older (n = 507) with the use of propensity scores to control for confounding bias. Propensity scores analysis for the non-elderly to the elderly were derived from a logistic regression (dependent variable: non-elderly vs. elderly group) taking into account following predictor variables: physician attitude or friendliness, male gender, type of physician visit, waiting time to see physician, and actual visit time spent with physician.

All statistical analyses were performed using the SAS (V9) statistical software [11]. Pearson's correlations were estimated to examine significant univariate predictors of patient satisfaction in both the elderly and non-elderly groups for physician satisfaction ratings. Some of the predictor variables examined for correlation included caring attitude/friendliness, first visit, routine reason for visit, male gender, waiting time, and time spent with physician. Variables significant at the univariate level were entered into a multivariate partial correlations analyses model to examine correlates of physician and office visit satisfaction adjusted for each others effects [4]. The significance level was set at the 0.05 level for all analyses.

## Results

The study sample was comprised of 507 elderly (age ≥ 65 years) and 507 non-elderly respondents. Detailed descriptive characteristics of the sample population are given in Table 1. On a scale of 0–100 (highest), the mean satisfaction with physician score was 82.72 for elderly respondents, which was significantly different from the mean score of 73.73 for non-elderly respondents (p < 0.001). Although they differed in physician ratings, pair-matched elderly patients and non-elderly patients reported similar waiting times (22.7 minutes for the elderly vs. 23.7 minutes for the non-elderly), and almost identical visit times (12.21 minutes for the elderly vs. 12.15 minutes for the non-elderly, p < 0.05). The elderly included the same percentage of male respondents (45.2% for both groups), but a greater percentage reported a first time visit to the physician (19.1% vs. 15.4% for the non-elderly, p < 0.001). However the elderly patients had a higher percentage reporting a visit for a routine exam or check-up (34.9% vs. 31.6% for the non-elderly, p < 0.05)

**Table 1:** Baseline characteristics of the study population (unmatched comparison of elderly vs. non-elderly group)

**Parameter** ⇓	**Total sample** **(N = 1014)** **Mean (SD)**	**Non-elderly Respondents** **(N = 507)** **Mean (SD)**	**Elderly Respondents** **(N = 507)** **Mean (SD)**
Physician Satisfaction Score [0–100] §§§	78.22 (32.75)	73.73 (35.27)	82.72 (29.38)
Age group (%)			
18 – 24	10.55%	21.10%	-
25 – 34	10.55%	21.10%	-
35 – 44	22.68%	45.36%	-
45 – 64	6.21%	12.43%	-
65 +	50.00%	-	100%
Male Gender (%)	45.17%	45.17%	45.17%
First visit to office (%)	17.26%	15.38%	19.13%
Routine exam or check-up	33.23%	31.56%	34.91%
Wait Time	23.21(19.05)	23.73 (18.92)	22.69 (19.19)
Less than 15 min	40.73%	37.87%	43.59%
15 to 30 min	38.95%	41.42%	36.49%
30 to 60 min	12.82%	13.21%	12.43%
60 min +	7.50%	7.50%	7.50%
Visit Time	12.18 (4.24)	12.15 (4.23)	12.21 (4.26)
Less than 5 min	7.69%	7.50%	7.89%
5 to 10 min	24.75%	25.44%	24.06%
10 min +	67.55%	67.06%	68.05%

Note: paired t-tests comparing means in the elderly vs. non-elderly were performed ^§§§ ^p-value < .001.

Table 2 presents results of the partial rho correlations (adjusted for confounders) and Pearson correlations (unadjusted) of predictor variables with the corresponding satisfaction ratings for the physician. Shorter waiting time was more significantly associated with better treatment satisfaction in non-elderly patients (partial rho = -0.25 in the non-elderly compared to partial rho = -0.11 in the elderly, p < 0.05 for both correlation differences and correlation with satisfaction). Increased time spent with physician was significantly more associated with better physician satisfaction in the non-elderly patients (partial rho = 0.38 in the non-elderly compared to partial rho = 0.18 in the elderly, correlation with satisfaction at p < 0.001 for both correlation differences and correlation with satisfaction).

**Table 2:** Partial correlates of physician satisfaction ratings in the study sample (N = 1014)

**Dependent Variable** → **Predictor Variables** ↓	**Physician Satisfaction** **N = 507**		**Physician Satisfaction** **N = 507**	
	**Age = < 65**		**Age 65+**	

	Partial§§ Correlation	Pearson§ Correlation	Partial§§ Correlation	Pearson§ Correlation

Caring Attitude/Friendliness	0.89***	0.89***	0.92***	0.96***
First Visit	-0.14**	-0.16***	-0.035	-0.19***
Routine Reason for Visit	0.14**	0.12**	0.083	0.096*
Male Gender	-0.019	0.014	-0.013	0.075
Waiting Time	-0.25***	-0.41***	-0.11*	-0.45***
Time Spent with Physician	0.38***	0.55***	0.18***	0.47***

Note: *** p-value < .001, ** p-value < .01, * p-value < .05. § Pearsons correlation used for univariate correlation estimations and §§ partial rho correlations used for multivariate correlation estimations.

In both the elderly and non-elderly groups, results showed that "friendliness" or "empathy" were highly correlated with physician satisfaction (partial rho = 0.92 in the elderly and partial rho = 0.89 in the non-elderly, both significantly correlated at p < 0.001 with physician satisfaction score). While no effects of gender or routine reason for visit were observed in the multivariate analyses, routine reason for visit to the physician was significantly correlated only in non-elderly patients with physician satisfaction scores (partial rho = 0.14, p < 0.01).

## Discussion

Quality of physician care is especially important in the elderly population, as high quality medical care can prevent hospitalization due to chronic conditions [12]. Insuring high quality of care is therefore an important goal to increase quality of life for those over 65 as well as decrease burden on the health care system. Physicians can enhance patients' perceptions of the quality of care by understanding the differences in perception and assessment of medical care that exist between the general and older population.

**Figure 1:**
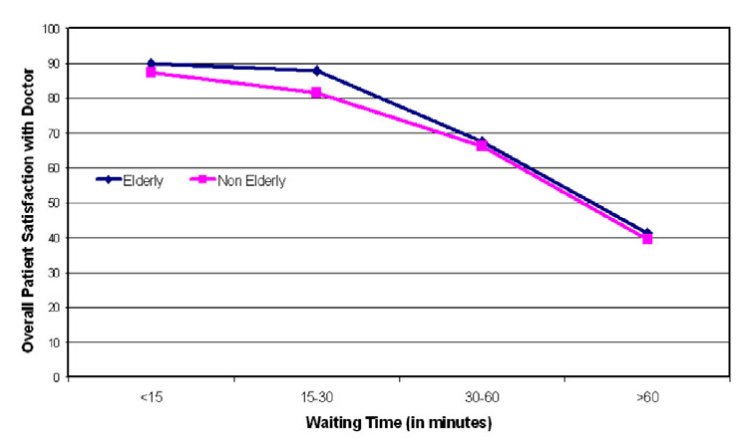
Relationship between waiting time and patients' satisfaction as a function of age.

Consistent with theory [13], we found that the caring attitude of the physician is a strong predictor of patient satisfaction. We also confirmed that waiting time and time spent with physician play key roles in the physician rating and satisfaction. Additionally, our study elucidated some of the differences between trends in satisfaction between the elderly and non-elderly. One of the most significant differences between these two groups exists in the time spent with physician and waiting time variables. To illustrate this, Figure 1 is shown to present the relationship between waiting time and patient satisfaction as a function of age. Initially, as waiting time increased, patients over the age of 65 were more forgiving than the younger group. One possible explanation of this difference may be that older patients are simply more likely to give higher satisfaction ratings, as numerous studies have shown [14-16]. However it doesn't explain why this trend does not continue as wait time increases; above 30 minute waits, overall patient satisfaction decreased for all age groups. It may be that for non-elderly patients, waiting time is more important when rating their physician. This is reflected in the stronger negative correlation of waiting time to physician satisfaction for the non-elderly as compared to the elderly patients.

Physician satisfaction also seems to be associated moderately and significantly with patient-reported time spent with the physician in the elderly but not nearly as strongly correlated with satisfaction as in the non-elderly. Other studies [17,18] have shown that increasing the visit time may provide physicians with a way to minimize and offset patient dissatisfaction when long waiting times is unavoidable. In following with our results, this may be more effective in the general population than in the older population.

All of these findings raise some important issues which deserve further study, for example: (1) even though elderly patients and non-elderly patients report similar waiting times, why do the elderly patients report overall higher satisfaction scores or why does increased waiting time seem to adversely affect satisfaction less in elderly patients; and (2) why does increased time spent with physicians not positively affect physician satisfaction in this group nearly as strongly as in the non-elderly? One could speculate that physicians are spending enough time with elderly patients, but the issue of waiting in physician offices needs further attention. The ways in which longer physician wait times could differentially affect older adults also may need further study and investigation.

A recent study found that the general practitioner's age was "negatively associated with patients' evaluation of all aspects of age, except accessibility" [19]. This is an especially interesting finding given that our study focused on the age of the patient rather than the physician. Possible future evaluations are needed to explore this new finding, perhaps taking into account both the age of the physician and patient and evaluating its effects on satisfaction ratings.

Quality of care is clearly a complex, multi-faceted concept. Thus it must be noted that our exploratory analysis had several limitations. First, a cross-sectional study does not permit causal inferences about the results. Second, our findings may be subject to respondent bias, since elderly patients may be less familiar with the use of the internet to rate their quality of medical care. Furthermore, self-reported data is subject to respondent recall bias and may have affected the survey responses we received, especially from the elderly group. Third, we did not measure variables such as race [7,14,15], health status [14,16,20,21], method of insurance [15,20], or patient trust of physician [4], which have been identified as correlates or possible correlates for patient satisfaction. Additionally many other factors may have influenced patient satisfaction ratings (such as accessibility, level of physician communication clarity, and patient expectations of the visit) but were not measured. Finally, satisfaction ratings in older adults can be heterogeneous; patients 65 to 69 tend to give higher ratings, while those 80 and older tend to give lower ratings [14-16,22]. Therefore, our findings may be affected by potentially greater social desirability bias (patients ranking physician highly because that is the socially desired norm) in elderly respondents.

## Conclusion

Despite the above limitations, this exploratory study provides further insight into the nature of patient satisfaction, particularly among the elderly. This study shows how elderly people rate aspects of care differently than the general adult population. It also presents areas of future improvement and study on the topic of patient satisfaction. Reducing wait times to see the physician could potentially be one of the key factors in improving patient satisfaction and associated patient care quality in elderly patients.

## Competing interests

Ms. Kong, Dr. Balkrishnan, and Dr. Anderson have no conflicts of interest to declare. Dr. Feldman and Mr. Camacho own equity in the Medical Quality Enhancement Corporation, which developed the Drscore.com website. This study was not funded by any source.

## Authors' contributions

Ms. Kong took the lead in the preparation of the manuscript and participated in the data analysis. Drs. Feldman, Anderson, and Mr. Camacho participated in the data collection, analyses, and critical revision of the manuscript. Dr. Balkrishnan conceived the paper, took the lead in performing the analyses, and participated in writing the manuscript and critically revising it.
